# Array-Based *FMR1* Sequencing and Deletion Analysis in Patients with a Fragile X Syndrome–Like Phenotype

**DOI:** 10.1371/journal.pone.0009476

**Published:** 2010-03-05

**Authors:** Stephen C. Collins, Brad Coffee, Paul J. Benke, Elizabeth Berry-Kravis, Fred Gilbert, Ben Oostra, Dicky Halley, Michael E. Zwick, David J. Cutler, Stephen T. Warren

**Affiliations:** 1 Department of Human Genetics, Emory University School of Medicine, Atlanta, Georgia, United States of America; 2 Joe DiMaggio Children's Hospital, Hollywood, Florida, United States of America; 3 Departments of Pediatrics and Neurological Sciences, Rush University Medical Center, Chicago, Illinois, United States of America; 4 Department of Pediatrics, Weill Cornell Medical College, New York, New York, United States of America; 5 Department of Clinical Genetics, Erasmus University, Rotterdam, The Netherlands; 6 Departments of Pediatrics and Biochemistry, Emory University School of Medicine, Atlanta, Georgia, United States of America; Brigham and Women's Hospital, Harvard Medical School, United States of America

## Abstract

**Background:**

Fragile X syndrome (FXS) is caused by loss of function mutations in the *FMR1* gene. Trinucleotide CGG-repeat expansions, resulting in *FMR1* gene silencing, are the most common mutations observed at this locus. Even though the repeat expansion mutation is a functional null mutation, few conventional mutations have been identified at this locus, largely due to the clinical laboratory focus on the repeat tract.

**Methodology/Principal Findings:**

To more thoroughly evaluate the frequency of conventional mutations in FXS-like patients, we used an array-based method to sequence *FMR1* in 51 unrelated males exhibiting several features characteristic of FXS but with normal CGG-repeat tracts of *FMR1*. One patient was identified with a deletion in *FMR1*, but none of the patients were found to have other conventional mutations.

**Conclusions/Significance:**

These data suggest that missense mutations in *FMR1* are not a common cause of the FXS phenotype in patients who have normal-length CGG-repeat tracts. However, screening for small deletions of *FMR1* may be of clinically utility.

## Introduction

Fragile X syndrome (FXS) is an X-linked dominant disorder that is the most frequently encountered form of inherited intellectual disability. In 1991, the common causal mutation in FXS was identified to be a large CGG trinucleotide repeat expansion in the 5′-untranslated region of the gene *FMR1*, the so-called full mutation [1]. Shortly thereafter, several groups identified *FMR1* deletions in FXS patients, suggesting that multiple mutational mechanisms could give rise to the disorder [2], [3], [4], [5]. The subsequent identification of an I304N *FMR1* missense mutation in a severely affected FXS patient suggested that yet another class of *FMR1* mutation was potentially a significant cause of disease [6]. However, while both trinucleotide repeat expansion [7] and *FMR1* deletions [8] have proven to be the usual basis of FXS, no additional missense mutations have been identified in the subsequent 17 years.

Several groups have previously attempted to identify additional *FMR1* missense mutations in patients without the full mutation but presenting with an FXS-like phenotype [9], [10], [11], [12], [13]. However, these previous studies were mutational screens and not designed to comprehensively evaluate the frequency of *FMR1* missense mutations in FXS. Three of the studies surveyed fewer than ten FXS-like patients [9], [10], [12], while the other two studies used less proven detection methods to survey only a portion of the *FMR1* coding sequence [11], [13]. There is a lack of case reports and clinical studies detailing individuals with coding changes in *FMR1* since *FMR1* sequencing is rarely performed in the clinical setting. Thus, the frequency of such mutations responsible for a FXS clinical picture is not known.

In this study, we used array-based resequencing to search for missense mutations in *FMR1* in a population of 51 unrelated FXS-like males. Despite achieving a high level of sequence coverage and accuracy, we did not identify any missense variants in *FMR1*, nor did we identify any novel noncoding variants likely to have a functional effect. Our method did, however, identify a pathogenic *FMR1* deletion in a patient with FXS.

## Methods

### Subjects and Samples

This study was approved by the Emory University Institutional Review Board (IRB ID: 1317–2004). All patients and/or legal guardians gave written informed consent to participate in this study. We recruited 51 unrelated intellectually disabled males who previously tested negative for the *FMR1* full mutation (>200 CGG repeats) and exhibited at least two of the FXS-like features listed in Table 1. Forty-seven of the patients were of European descent and four were of African descent. A focused clinical history and either a blood or saliva specimen were obtained from each patient. DNA was extracted from the obtained specimens using standard methods as were isolation of lymphoblastoid cells from whole blood.

**Table 1:** Phenotypic characteristics of FXS-like patients.

Characteristic	Examples
FXS-like facial features	Elongated face, everted ears, macrocephaly
Macroorchidism	
Connective tissue abnormalities	Hyperextensible finger joints, velvety skin, or recurrent ear infections
Shyness or poor eye contact	
Attention deficit/hyperactivity	
Language delay	
Repetitive behaviors	Hand flapping, hand biting
Evidence of X-linked inheritance	Similarly affected male sibling, affected second-degree male relative through maternal lineage

Patients enrolled as FXS-like exhibited at least two of these characteristics.

### *FMR1* Sequencing

**Figure 1:**
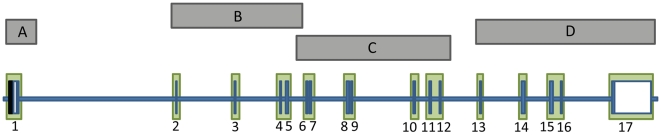
Targeted resequencing of *FMR1*. The horizontal axis is formed by intronic sequence, and the numbered vertical spokes represent the 17 exons of *FMR1*. Coding exonic sequence is shown in blue, while noncoding exonic sequence is shown in white. The black region upstream of exon 1 is the minimal promoter of *FMR1*. The grey bars represent the four LR-PCR amplicons used for sequencing. The green boxes represent the *FMR1* regions sequenced with the custom resequencing array.

*Targeting FMR1*. Four long range PCR (LR-PCR) amplifications were designed to target *FMR1* (Figure 1). The LR-PCR primer pairs were as follows: *FMR1*A-F: 5′-CAGACTGCGCTACTTTGAACC-3′ and *FMR1*A-R: 5′- CTACATACCAACAAACGCACTACTGCTACAT-3′; *FMR1*B-F: 5′- AATTTCCAGTATACTTGTCTATTTTTCGAGATG-3′ and *FMR1*B-R: 5′- TTTTGGGAGATAGCTACCTACAGGGTATCTGATT-3′; *FMR1*C-F: 5′- GTTGAACATTAAATTGCAGTTCAGAATACATAG-3′ and *FMR1*C-R: 5′- GAGACATATCCAATCCACTTGCCGTTATAGT-3′; *FMR1*D-F: 5′- AATAATCTGATACGTTTAAAAGGTTGCTATTGA-3′ and *FMR1*D-R: 5′- TTAATATGGTTTAGTGGCACCCTATGTAATAAA-3′. Each LR-PCR-A reaction contained 50 ng of genomic DNA, 100 ng of each primer, 5 µl of dNTPs (Takara Bio Inc., Otsu, Shiga, Japan), 12.5 µl of 2x GC Buffer II (Takara), and 0.5 µl of Ex Taq (Takara), in a total of 25 µl. The following PCR conditions were used for LR-PCR-A: initialization at 95°C for 4 minutes; 37 cycles of denaturation at 95°C for 30 seconds and annealing/elongation at 60°C for 4 minutes; and a final elongation step of 72°C for 9 minutes. Each LR-PCR-B, -C, and -D reaction contained 50 ng of genomic DNA, 100 ng of each primer, 4 µl of dNTPs (Takara), 2.5 µl of Ex Taq Buffer (Takara), and 0.4 µl of Ex Taq (Takara), in a total of 25 µl. The following PCR conditions were used for LR-PCR-B: initialization at 94°C for 4 minutes; 30 cycles of denaturation at 94°C for 20 seconds and annealing/elongation at 64°C for 8 minutes; and a final elongation step of 68°C for 13 minutes. The same conditions were used for LR-PCR-C, but 35 cycles of denaturation and annealing/elongation were used instead of 30. The same conditions used for LR-PCR-C were used for LR-PCR-D, but the annealing/elongation at 64°C was continued for 9 minutes instead of 8 minutes. The expected sizes of the LR-PCR amplicons were confirmed by gel electrophoresis.

*Sequencing-by-hybridization*. *FMR1* sequencing was performed on Custom Resequencing Arrays (Affymetrix, Santa Clara, CA), designed to provide coverage of all 17 *FMR1* exons and the *FMR1* promoter, plus 200 bp of flanking intronic sequence (Figure 1). Patient sample amplicons were processed for sequencing-by-hybridization according to the Affymetrix CustomSeq Resequencing Array protocol, Version 2.1, with the following exceptions. The four LR-PCR amplicons per patient were pooled in equimolar fashion to a total of 4 µg and digested with 0.2 units of DNase I (Promega, Madison, WI) at 37°C for 3 minutes, yielding digestion products between 100–600 bp. Labeling, hybridization, and array processing were performed as per the protocol.

*Variant Detection and Confirmation*. Base-calling was performed with the ABACUS statistical method [14] using the POPGEN genotyping software [15]. Putative variants were confirmed by traditional Sanger sequencing of fresh LR-PCR amplicons. Both POPGEN data and DNA chromatograms were inspected manually with the SeqScape software (Applied Biosystems, Foster City, CA).

### Western Blotting

Immunoblotting was performed using standard methods. Briefly, patient and control lymphoblastoid cells were lysed with a standard Triton X-100-based lysis buffer. The lysate protein concentrations were measured with the Bradford assay. Proteins were denatured by heating at 95°C for 3 minutes and separated by polyacrylamide gel electrophoresis and transferred to a nitrocellulose membrane. To assess protein loading and transfer, the membrane was reversibly stained with Ponceau S. The membrane was blocked for one hour in blocking buffer (10 g dry milk, 200 µl Tween-20, and 100 ml PBS), probed with primary antibody (anti-FMRP 1a or anti-eIF4e) overnight, and probed for one hour with horseradish-peroxidase conjugated anti-mouse secondary antibodies. Proteins were detected by chemiluminescence (ECL, GE Healthcare, Piscataway, NJ).

## Results

### Sequence Accuracy

Across the 51 FXS-like patients sequenced by array hybridization, 99.6% of bases were called with high reliability, as determined by a quality score of 30 or greater. The high level of sequence accuracy is further demonstrated by the identification of known polymorphisms. As seen in Table 2, we detected all seven SNPs catalogued in dbSNP (build 130) for which the population frequency has been measured in HapMap samples. For the sake of comparison, we weighted the HapMap frequency data by the racial distribution of our patient population. None of the SNPs were found to be at a statistically different frequency in the FXS-like patients from in the HapMap controls, suggesting that the *FMR1* resequencing arrays reliably detect sequence variants.

**Table 2:** Detection of known polymorphisms in FMR1 by array resequencing.

SNP	FXS-like patient frequency	Weighted HapMap frequency	p-value
rs25726	0.176	0.073	0.23
rs25731	0.078	0.062	1
rs25707	0.137	0.072	0.53
rs29281	0.039	0.007	0.50
rs25714	0.078	0.084	1
rs29285	0.039	0.007	0.50
rs25704	0.353	0.280	0.52

P-values reflect the result of Fisher's exact tests.

### Novel *FMR1* Sequence Variants

Notably, no novel variants were detected in the *FMR1* coding sequence in the population of 51 FXS-like males. However, two novel intronic variants, c.52-47A>G and c.105-179G>T, were identified in *FMR1* (Table 3). As an assessment of possible functional relevance, we examined the mammalian conservation of these nucleotide positions and their genomic regions using phyloP and phastCons scores, respectively [16]. Because both variants are located in poorly conserved genomic regions (phastCons of 0.01), it is likely that they represent rare neutral variants that lack functional significance.

**Table 3:** Novel FMR1 sequence variants identified in FXS-like males.

Location	cDNA Variant	PhastCons	PhyloP	Patient Frequency
Intron 1	c.52-47A>G	0.01	1.27	1/51
Intron 2	c.105-179G>T	0.01	1.06	1/51

### Array-Based Deletion Detection

**Figure 2:**
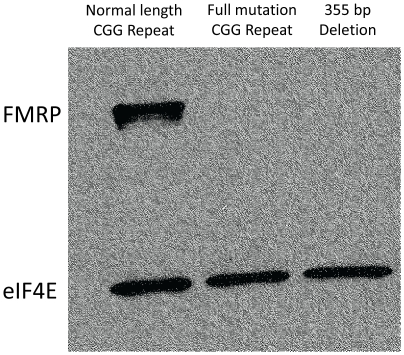
FMRP expression in control and fragile X tissues. Western blot of lymphoblastoid cell lysate from a healthy control, a fragile X patient, and a patient harboring a novel deletion in the 5′UTR of *FMR1*. The protein eIF4e was assessed as a loading control.

In addition to detecting point mutations, resequencing arrays allow the detection of deletions. In one FXS-like patient, we identified a 355 bp deletion extending from 220 bp upstream of the CGG repeat through the second codon of the *FMR1* coding sequence (i.e. hg18, chr.X: 146801041–146801395). After confirming this deletion with Sanger sequencing, we assessed its effects on FMRP translation. As shown in Figure 2, immunoblot analysis of patient lymphoblastoid cell line lysates revealed an absence of FMRP expression.

## Discussion

We have sequenced the promoter, exons, and splice junctions of *FMR1* in 51 unrelated patients with several classic features of FXS but without the full mutation utilizing resequencing arrays. Two novel intronic variants were identified which likely have no functional effect. Notably no missense or promoter mutations were found. As the largest sequencing analysis of FXS-like patients to date, these data suggest that *FMR1* sequence variants are not a significant cause of the FXS phenotype.

At the present time, two missense changes in *FMR1* have been identified, the benign and polymorphic p.A145S variant (rs29281) and the p.I304N mutation previously detected in a severely affected FXS-like patient [6]. It is surprising that these are the only missense changes that have been found in *FMR1*. In comparison, over 100 distinct point mutations in the nearby gene *MECP2* have been shown to cause Rett syndrome, despite the fact that the gene is smaller and more recently identified than *FMR1* [17]. Furthermore, because a functional absence of the *FMR1* gene product is compatible with life, albeit associated with the symptoms of FXS, missense changes in *FMR1*, which in many cases would be less damaging than a loss-of-function, should not lead to embryonic lethality.

Since there is no reason to assume the *FMR1* gene is less mutable than any other gene, why are conventional mutations uncommon among patients presenting with FXS-like features but without the full mutation? There are several possible explanation for absence of missense mutations. First, unlike Rett syndrome or many other Mendelian syndromes, the phenotype of FXS is subtle and variable. This makes a firm clinical diagnosis often difficult, even for an experienced clinician. Second, many syndromic aspects of FXS individually are not unusual in a developmentally delayed male population (i.e. language delay) and our criteria of only two features (Table 1) for study inclusion may have been too lenient. Third, it is possible that the phenotypic consequence of missense mutations might be distinct from classic FXS, leading to a more subtle isolated developmental/behavioral phenotype, such nonspecific intellectual disability, or even autism, learning disability, anxiety disorder or attention-deficit/hyperactivity disorder, without overall intellectual disability. Similarly, a *FMR1* missense mutation could selectively alter the function of only one domain of FMRP, thereby causing a specific FXS-like symptom, such as connective tissue defects or macro-orchidism, in the absence of an overall FXS-like phenotype. Given the already high level of genetic heterogeneity among patients with developmental disability [12], [18], [19], this heterogeneity may be further compounded by any of these possibilities. Perhaps accepting the unavoidable heterogeneity and sampling a much larger cohort with minimal clinical criteria (i.e. diagnostic laboratory samples submitted to “rule out FXS”) would be profitable. While much more costly, recent advances in sequencing-by-synthesis may allow such studies.

The current study confirms the known importance of occasional *FMR1* deletions responsible for FXS. The deletion we identified extends from 220 bp upstream of the CGG repeat through the second codon of the *FMR1* coding sequence, and results in the absence of FMRP expression in patient tissues. While the exact breakpoints are unique, this deletion belongs to a well-characterized class of deletions that result from the instability of the CGG trinucleotide repeat region [8], [20]. This deletion, as a null mutation, would be expected to present with a FXS phenotype as the *FMR1* full mutation is also a functional null mutation. Since *FMR1* deletions are not specifically screened for clinically and are usually found secondary to CGG-repeat screening, many small deletions and perhaps duplications may be missed in routine testing of patients with a FXS presentation. Therefore screening for small *FMR1* copy number variation might be clinically useful and could be accomplished by targeting *FMR1* for high density coverage in clinical arrays screened by comparative genome hybridization
